# Complicated Isolated Liver Abscess Caused by Viridans Group Streptococci Leading to Right Hepatectomy

**DOI:** 10.7759/cureus.9149

**Published:** 2020-07-12

**Authors:** Muhammad F Ahmed, Zainab Abbasi, Sajan Das, Alok Aggarwal, Sonu Sahni

**Affiliations:** 1 Internal Medicine, Brookdale University Hospital Medical Center, Brooklyn, USA; 2 Internal Medicine, Liaquat University of Medical and Health Sciences, Jamshoro, PAK; 3 Internal Medicine, Kingsbrook Jewish Medical Center, Brooklyn, USA; 4 Surgery, Brookdale University Hospital Medical Center, Brooklyn, USA; 5 Research Medicine, New York Institute of Technology College of Osteopathic Medicine, New York, USA; 6 Primary Care, Touro College of Osteopathic Medicine, New York, USA

**Keywords:** pyogenic liver abscess, streptococcus viridans, hepatectomy, ultrasound-guided drianage

## Abstract

Pyogenic liver abscesses (PLAs) secondary to bacterial etiologies are most often seen in developing countries and are less common in North America. The predominant etiology is infection occurring in the setting of direct extension of hepatobiliary or intestinal infection. The most common pathogen isolated from a PLA in the United States is *Escherichia coli*, whereas *Streptococcus viridans* is a rare entity in the developed world. Herein we report a rare case of a complicated isolated PLA in a patient without any known comorbidities which lead to hepatectomy. The patient was born and raised in the United States with no recent travel history. The patient was found to have 10 cm isolated multicystic mass on imaging confirmed later as vancomycin-resistant *Streptococcus viridans* PLA. The patient was treated with multiple intravenous antibiotics and underwent multiple ultrasound-guided percutaneous abscess drainages by interventional radiology, but all unsuccessful. The patient underwent right posterior liver lobectomy, thereafter making a quick recovery and was discharged. Our case underlines the significance of considering liver abscess as a differential even in previously healthy individuals with no known prior comorbid conditions, as prompt recognition is imperative in preventing morbidity and mortality.

## Introduction

Viridans group streptococci (VGS) are a large group of commensal bacteria, most commonly a part of human oral flora and live in close association with the gingiva and the teeth. These catalase-negative gram-positive organisms are further divided into five groups: *Streptococcus mitis* group, *Streptococcus*​​ ​​​*anginosus* group, *Streptococcus ​​​​​​*​*bovis* group, *Streptococcus mutans* group, and *Streptococcus *​​​​​​​*salivarius* group [1]. Each of these groups potentially leads to a distinct disease presentation varying from bacteremia, abscess formation to endocarditis if introduced into the bloodstream. Pyogenic liver abscess (PLA) is a liver abscess caused by bacterial infection, most commonly by intestinal flora. It is most often seen in seen in developing countries and is less commonly seen in North America with an incidence of approximately 2.3 cases per 100,000 [2]. VGS are now being increasingly recognized as an opportunistic disease-causing organism in an immunocompromised host but rarely presents in PLA [3]. The pathomechanism of PLA is that infection which occurs in the setting of direct extension hepatobiliary or intestinal infection and hematogenous spread [4]. Herein we report a unique case of an otherwise healthy individual with no known comorbid conditions that presented with a large pyogenic hepatic abscess caused by VGS that remained resistant to conventional medical and interventional management strategies and ultimately right hepatectomy was performed as a curative treatment.

## Case presentation

A 52-year-old African American male with no significant past medical history presented to the emergency department with a chief complaint of sudden onset of right-sided abdominal pain that continued to worsen for a few days prior to presentation. On day of presentation, the patient stated that the pain had become excruciating rated 10/10 in severity which he described as dull in nature, non-radiating with no aggravating or relieving factors. He also complained of associated right-sided chest and flank pain. He reported subjective fevers but denied any chills, nausea, vomiting, diarrhea, measured weight loss, or any recent travels outside of the United States. Social history was insignificant.

On presentation, the patient was noted to be tachycardic with a fever of 102.5°F (39.2°C), respiratory rate of 20 breaths per minute, saturating at 99% on room air, and blood pressure of 115/65 mmHg. He was noted to be alert, awake, oriented, and was in mild distress. Ocular examination showed icterus. Throat and neck examination were completely benign, with no oral lesions or lymphadenopathy. Chest examination revealed normal heart sounds, and lung sounds were also normal and vesicular. His abdomen was soft and distended with tenderness noted in all quadrants. Murphy’s sign was negative. Bowel sounds were normal in all quadrants. No masses were palpated otherwise.

Laboratory studies demonstrated hemoglobin of 12.2 g/dL, white blood cell (WBC) count of 12.10 x 10^9^/L with 77.1% neutrophils, and platelets of 137 x 10^9^/L. The metabolic panel revealed normal blood urea nitrogen and creatinine levels. However, liver enzymes were elevated with an alanine aminotransferase (ALT) level of 237 U/L, aspartate aminotransferase (AST) 236 U/L, alkaline phosphatase of within normal range, total bilirubin 1.8 mg/dL, and lactate was normal at 1.4 mmol/L. The patient was also tested for hepatitis B and C markers, which came out to be negative. His initial laboratory findings are summarized in Table 1.

**Table 1:** Initial Laboratory Data L, Low; H, High

	Results	Reference Range
White Blood Cell Count	12.10	4.10-10.10 × 10^9^/L
Red Blood Cell Count	5.45	4.33-5.43 × 10^12^/L
Hemoglobin	12.2	13.4-15.4 g/dL
Hematocrit	37.9	40.0%-47.0%
Mean Corpuscular Volume	69.4	80.8-94.1 fL
Platelet Count	137 (L)	153-328 × 10^9^/L
Neutrophils Absolute	9.40 (H)	1.40-6.80 × 10^9^/L
Lymphocytes Absolute	0.40 (L)	1.10-2.90 × 10^9^/L
Monocytes Absolute	2.30 (H)	0.20-1.00 × 10^9^/L
Eosinophils Absolute	0	0.00-0.40 × 10^9^/L
Basophils Absolute	0.10	0.00-0.10 × 10^9^/L
Prothrombin Time	21.8 (H)	9.2-12.8 seconds
International Normalized Ratio	1.91 (H)	0.70-1.20
Partial Thromboplastin Time	30.7	23.5-35.5 seconds
Glucose	132 (H)	70-99 mg/dL
Blood Urea Nitrogen	15	9.0-20.0 mg/dL
Creatinine	0.89	0.66-1.25 mg/dL
Sodium	140	133-145 mEq/L
Potassium	4.5	3.5-5.1 mEq/L
Chloride	106	98-107 mEq/L
Bicarbonate	28	22-30 mEq/L
Calcium	8.2 (L)	8.4-10.2 mg/dL
Anion Gap	11	8-12 mEq/L
Protein, Total	6.6	6.3-8.2 g/dL
Albumin	4.3	3.5-5.0 g/dL
Bilirubin, Total	1.8 (H)	0.2-1.3 mg/dL
Bilirubin, Direct	0.9 (H)	0.0-0.4 mg/dL
Alanine Transaminase	237 (H)	21-72 U/L
Aspartate Transaminase	236 (H)	17-59 U/L
Alkaline Phosphatase	124	38.0-126.0 U/L
Lipase	194	23-300 U/L
HIV Antibody 1 and 2	Negative	
Hepatitis B Surface Antigen	Negative	
Hepatitis C Antibody	Negative	

**Figure 1:**
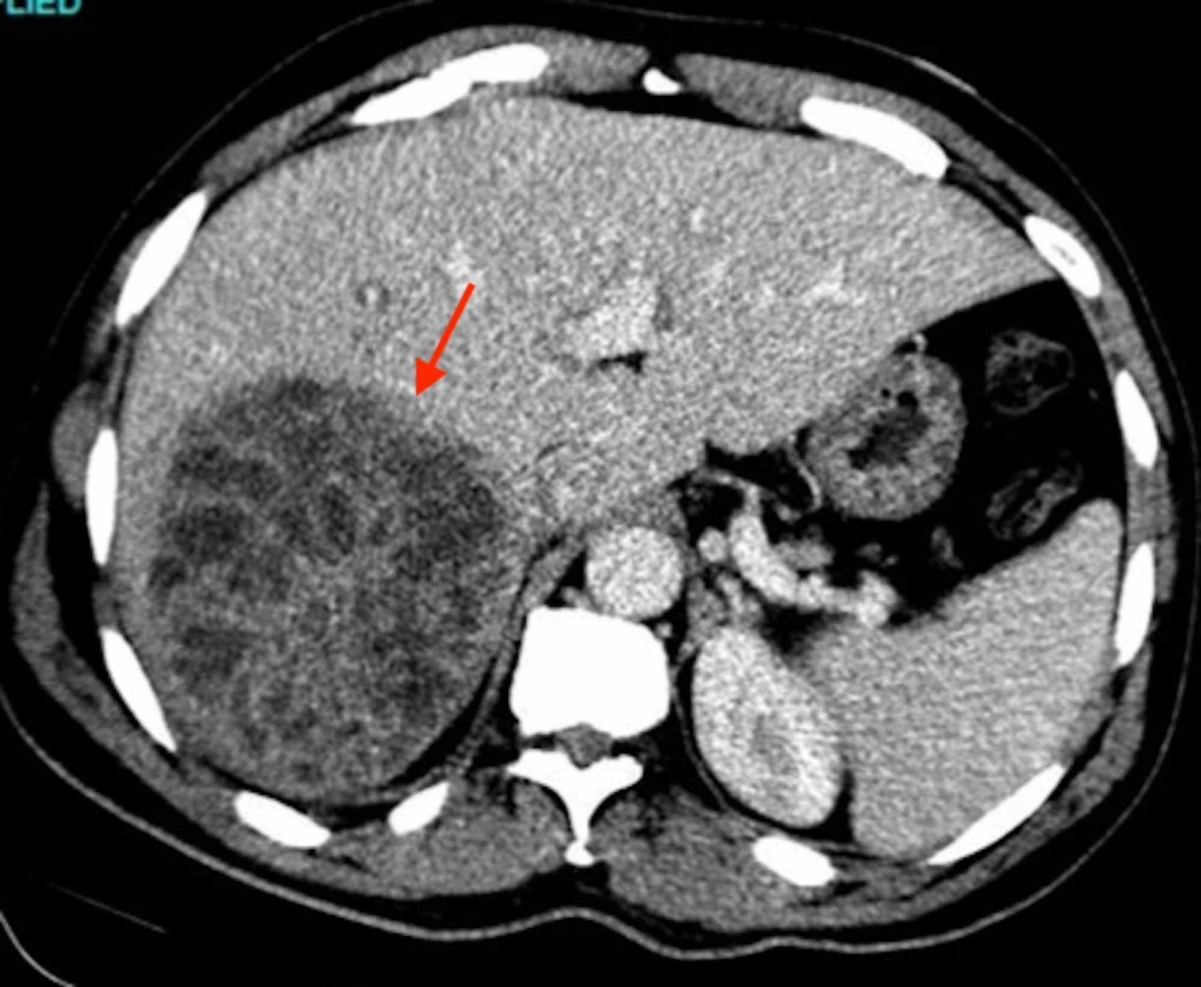
CT of the abdomen demonstrating a large 10 cm heterogeneous hypodensity in the post right hepatic lobe likely a liver abscess. The arrow points to area of interest.

CT of the abdomen was performed with intravenous contrast, which demonstrated a large 10 cm heterogeneous hypodensity in the post right hepatic lobe with normal hepatic and portal veins and no internal gas loculations (Figure 1). It was deemed as a likely liver abscess with a mass being an unlikely possibility due to lack of biliary dilation and no mass effect.

**Figure 2:**
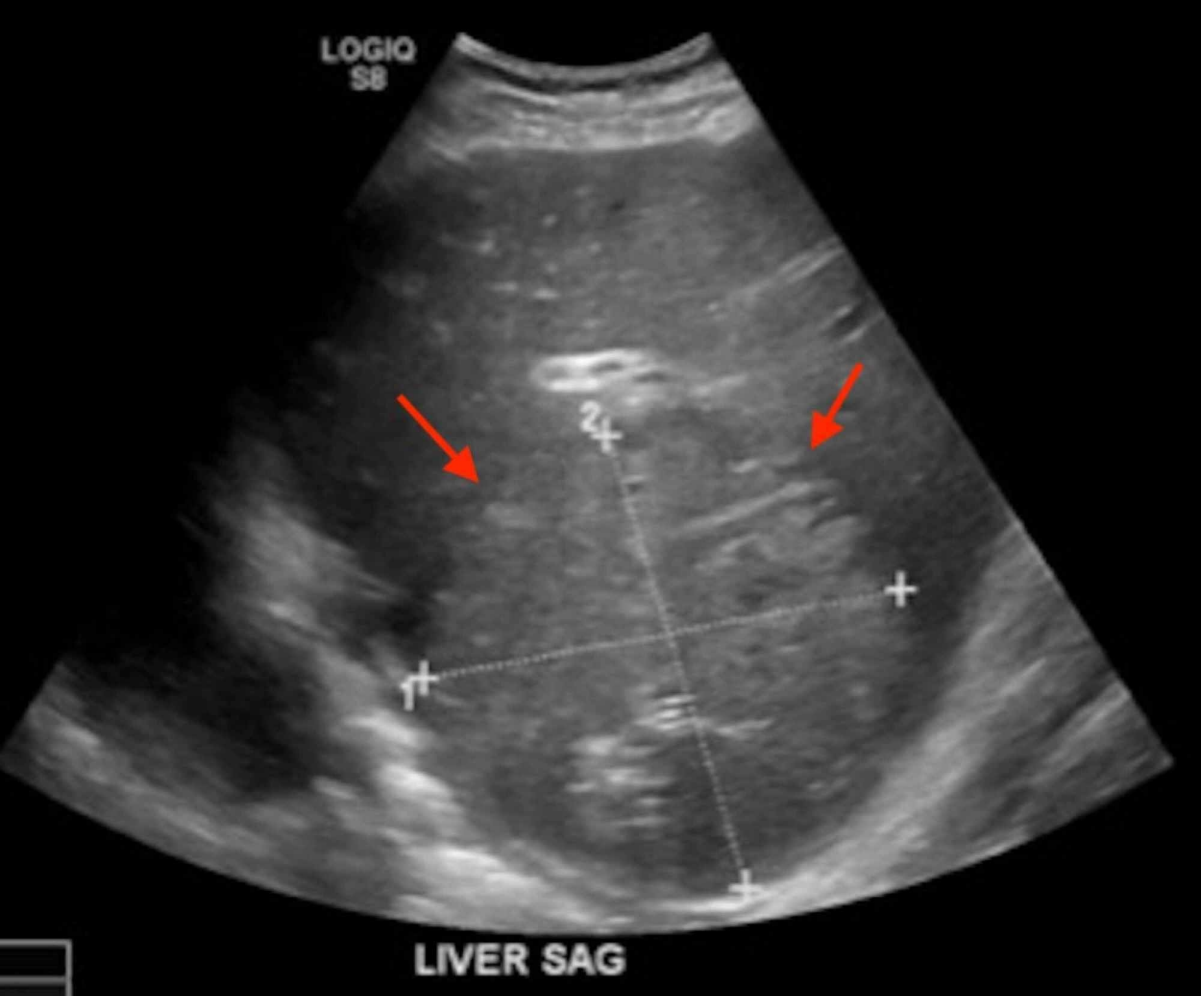
Ultrasonography of the abdomen demonstrating an increase in the size of the abscess to 12.3 x 11.9 cm after hepatic drain placement. The arrows point to area of interest.

Blood cultures drawn on the day of admission grew VGS on day 4 of admission, which was unable to grow for susceptibility testing. The patient was started on intravenous fluids for volume resuscitation and covered empirically with ceftriaxone and metronidazole. Ultrasound-guided hepatic drain placement was performed by interventional radiology, draining 100 mL of sanguinopurulent fluid. The aspirate fluid culture was also shown to be positive for VGS. The patient also underwent transthoracic echocardiography to rule out infectious endocarditis which did not reveal infective endocarditis. The patient was noted to have a persistently elevated international normalized ratio (INR) which peaked at 2.44 and was started on oral vitamin K supplementation uptitrated from 2.5 mg daily to eventually 10 mg which was continued daily. He later underwent ultrasonography of the abdomen on day 4 of hepatic drain placement to evaluate the abscess, which demonstrated an increase in the size of the abscess to 12.3 x 11.9 cm (Figure 2).

**Figure 3:**
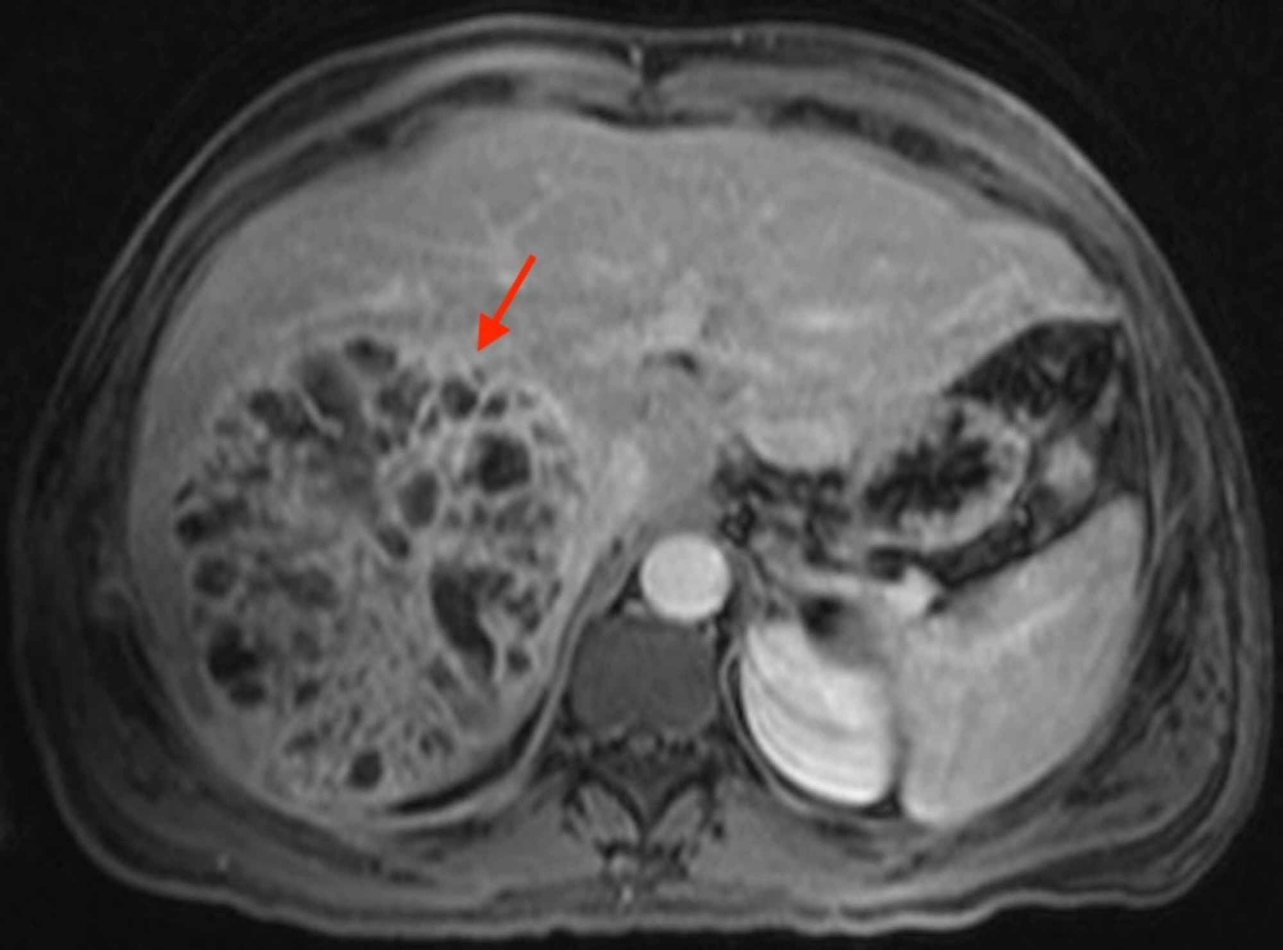
MRI of the abdomen demonstrating a 14.0 x 13.5 x 1.5 cm multicystic enhancing mass lesion within the segments 7 and 8 of the liver with an enhancing pseudocapsule. The arrow points to area of interest.

The patient subsequently underwent CT-guided placement of a large-bore pigtail catheter, and the hepatobiliary surgery department was consulted. It was recommended to obtain an MRI of the abdomen with the liver protocol, which showed a 14.0 x 13.5 x 1.5 cm multicystic enhancing mass lesion within the segments 7 and 8 of the liver with an enhancing pseudocapsule (Figure 3).

**Figure 4:**
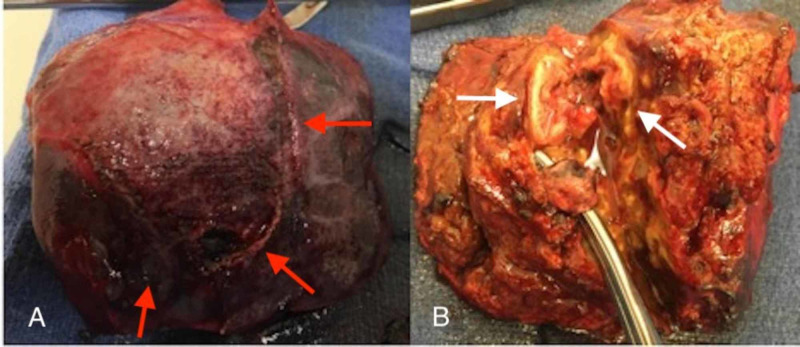
(A) Surgical specimen of right posterior liver involving segments 6, 7, and partially 8. (B) Liver specimen demonstrating an abscess cavity with multiloculated and multiple smaller pocket. The arrows point to areas of interest.

Numerous cystic components with enhancing walls were noted throughout the lesion. There was no infiltration within the hepatic parenchyma with non-displacement of vascular architecture. The remainder of the liver was normal in enhancement. Concerns for *Echinococcus* *granulosus* were ruled out with serological testing. Given the multiloculated nature of the abscess, failure of adequate drainage using large-bore pigtail catheter, and clinical worsening of the patient with concern for sepsis, the patient was transferred to surgical service. The surgical team carried out radiology-guided drainage which drained 20 mL of sanguinopurulent fluid but was unsuccessful for resolution. The aspirated fluid was noted to be positive for VGS resistant to vancomycin. With the patient continuously spiking fevers and failure of improvement with radiological drainage of the abscess, surgical drainage was planned. The patient underwent right posterior liver lobectomy and appendectomy. Intraoperative findings include an abscess cavity involving segments 6, 7, and partially segment 8 of the liver with multiloculated and multiple smaller pockets (Figure 4).

Intraoperative specimens were sent for histopathology. Postoperatively, the patient was observed in the surgical intensive care unit. Histopathology report showed benign hepatic parenchyma with extensive acute and chronic granulomatous inflammation with no signs for malignancy. Following surgery, the patient made a quick recovery and was discharged from the hospital. He continues to follow at the outpatient clinic and is clinically doing well.

## Discussion

PLAs are most often seen in developing countries and are less commonly seen in North America. The most common etiology is infection that occurs in the setting of direct extension hepatobiliary or intestinal infection and hematogenous spread [4]. Amongst the intra-abdominal causes, suppurative appendicitis is also a leading cause [5]. To our knowledge, this is the first reported case of a complicated PLA in a patient without any comorbidities which lead to hepatectomy .The most common pathogen isolated from a PLA in the United States is* Escherichia coli;* however, some studies indicate *Klebsiella* as well [6]. Members of the viridans group include *Streptococcus mitis, Streptococcus mutans, Streptococcus oralis, Streptococcus​​​​​​​ sanguinis, Streptococcus​​​​​​​ sobrinus*, and the* Streptococcus​​​​​​​ milleri* group. In one Canadian study of patients with PLA, *Streptococcus millieri* was the most often member of the viridans group which was identified [7]. Studies indicate an equal distribution of disease amongst males and females or a slightly higher prevalence in males as compared to females [8-10]. The pathogenic mechanism involves the failure of the initial inflammatory response to clear the insult to the liver. The classification of the abscesses is based on the route of infection which is via the hepatobiliary tree, portal vein, hepatic artery, a direct extension of infection from the contiguous area, and post-penetrating trauma to the liver [11].

Presenting symptoms include fever, chills, and abdominal pain. Laboratory abnormalities include leukocytosis (84% of patients), anemia (88.9% of patients), hypoalbuminemia (94% of patients), and an elevated alkaline phosphatase (73% of patients) [12]. Diagnosis is usually made by radiological imaging with ultrasonography or CT [13]. The mainstay of treatment remains antibiotics with percutaneous drainage by interventional radiology, which is effective in the majority of patients [6]. If left untreated, mortality rates of 80%-100% have been reported [12]. In a retrospective study of 96 patients with PLA, it was demonstrated that in liver abscesses more than 5 cm, surgical drainage provides better clinical outcomes than percutaneous drainage [14].

While in our case the patient was previously healthy, the majority of cases of PLA have been reported in patients in developing countries or who are immunocompromised or in patients who have underlying comorbidities such as diabetes and/or neoplasm. In our case, the patient presented with left-sided abdominal pain and weight loss. Labs were significant for leukocytosis, elevated ALT and AST >200 U/L, alkaline phosphatase, and prothrombin time/INR. The patient developed anemia and thrombocytosis, and had persistently elevated INR during the hospital course. The patient was treated with different intravenous antibiotics and underwent multiple ultrasound-guided percutaneous abscess drainages by interventional radiology, but all were unsuccessful. The patient's condition was not improving, and a repeat CT scan and MRI showed an interval increase in the size of abscess despite multiple drains placed. The surgical department was consulted as the patient was unresponsive to standard treatment modalities and was developing signs of sepsis. The patient was scheduled for right posterior lobectomy and appendectomy. Multiple units of fresh frozen plasma were transfused to correct INR prior to surgery. The patient’s condition improved after surgery. Abdominal pain improved, nausea resolved, and appetite improved. The patient kept following with the hematology and surgery clinics for elevated INR as an outpatient for postoperative follow-up. On his last clinical visit, his INR is normal and he is maintaining his weight.

## Conclusions

Hepatic abscesses are less common in North America. In the cases reported, most of them occur in people with preexisting medical conditions and respond to the standard treatment modality which includes a combination of antibiotics and percutaneous drainage. Our case underlines the significance of considering liver abscess as a differential even in previously healthy individuals with no known prior comorbid conditions. It also serves to indicate lobectomy as a sequela for patients who do not respond to the standard treatment modality.
